# Matrix Effects in GC–MS Profiling of Common Metabolites after Trimethylsilyl Derivatization

**DOI:** 10.3390/molecules28062653

**Published:** 2023-03-15

**Authors:** Elena Tarakhovskaya, Andrea Marcillo, Caroline Davis, Sanja Milkovska-Stamenova, Antje Hutschenreuther, Claudia Birkemeyer

**Affiliations:** 1 Department of Plant Physiology and Biochemistry, Faculty of Biology, St. Petersburg State University, 199034 St. Petersburg, Russia; elena.tarakhovskaya@gmail.com; 2 Vavilov Institute of General Genetics RAS, St. Petersburg Branch, 199034 St. Petersburg, Russia; 3 Mass Spectrometry Research Group, Faculty of Chemistry and Mineralogy, Leipzig University, 04103 Leipzig, Germany; a.marcillo.lara@fz-juelich.de (A.M.); caroline_davis@waters.com (C.D.);; 4 Institute of Energy and Climate Research (IEK-8), Forschungszentrum Jülich GmbH, 52428 Jülich, Germany; 5 Waters GmbH, 1130 Vienna, Austria; 6 Bioanalytics Research Group, Faculty of Chemistry and Mineralogy, Leipzig University, 04103 Leipzig, Germany; s.milkovska-stamenova@adversis-pharma.de; 7 AP Diagnostics GmbH, 04103 Leipzig, Germany; 8 German Center for Integrative Biodiversity Research (iDiv) Halle-Leipzig-Jena, 04103 Leipzig, Germany

**Keywords:** quantification, gas chromatography–mass spectrometry, metabolomics, signal suppression, signal enhancement, compound saturation

## Abstract

Metabolite profiling using gas chromatography coupled to mass spectrometry (GC–MS) is one of the most frequently applied and standardized methods in research projects using metabolomics to analyze complex samples. However, more than 20 years after the introduction of non-targeted approaches using GC–MS, there are still unsolved challenges to accurate quantification in such investigations. One particularly difficult aspect in this respect is the occurrence of sample-dependent matrix effects. In this project, we used model compound mixtures of different compositions to simplify the study of the complex interactions between common constituents of biological samples in more detail and subjected those to a frequently applied derivatization protocol for GC–MS analysis, namely trimethylsilylation. We found matrix effects as signal suppression and enhancement of carbohydrates and organic acids not to exceed a factor of ~2, while amino acids can be more affected. Our results suggest that the main reason for our observations may be an incomplete transfer of carbohydrate and organic acid derivatives during the injection process and compound interaction at the start of the separation process. The observed effects were reduced at higher target compound concentrations and by using a more suitable injection-liner geometry.

## 1. Introduction

One of the routine tasks in modern biochemical research is the analysis of biological samples of different origins using “omics”-techniques, such as metabolomics. Among the metabolite profiling techniques, gas chromatography coupled with low-resolution mass spectrometry (GC–MS) is a relatively inexpensive, robust, and mature technology [1,2]. The GC–MS approach features a particularly high analytical performance allowing for simultaneous analysis of hundreds of small molecular weight compounds with different chemical structures [3,4,5], and hence, it is a very effective and popular method of metabolite profiling of extracts from biological material [6,7]. However, the accurate analysis of such extracts with this powerful method still challenges the classical preconditions of a valid quantification.

Biological samples are typically very complex. They contain substances of different chemical classes, such as sugars, organic acids, amino acids, phenolic compounds, fatty acids, and more. In addition, each class may include dozens of representatives (e.g., some plant extracts may contain about 25 proteinogenic and non-proteinogenic amino acids). Sometimes, the molecules of the same class are easily distinguishable by their specific mass spectra (such as most amino acids); other times, they are not (e.g., aldoses), requiring baseline chromatographic separation for the successful quantification of such compounds. However, when such complex samples are analyzed, similar retention, respectively, incomplete separation of compounds is often observed [8].

Quantitative analysis requires the area of the compound’s selective ion to be proportional to the change in concentration of a (free) compound in the sample [9]. According to IUPAC, “the analytical sample can be considered to be the combination of an analyte and a matrix”, with the latter being the analytical sample excluding the analyte. Here, an *interferent* is a component of the matrix that embodies an influence quantity (VIM 2.52): when composites of a sample (i.e., *matrix compounds*) considerably interfere with the analysis of other substances (i.e., *target compounds*), it is called a *matrix effect*. Matrix interference may occur at any step of the analytical protocol for GC–MS metabolite profiling. This includes extraction, sample derivatization, injection, chromatographic separation, and finally, MS detection [10,11,12,13,14,15]. Commonly, the injected sample volume or extract concentration is increased to further improve the detectability of the target compounds until reaching the matrix interference limit or until column and liner contamination limit accurate quantification. Unfortunately, a prior investigation to minimize matrix effects is often neglected in applied metabolite profiling studies, though these effects may lead to considerable misrepresentation of the data [14]; very likely, the tedious evaluation of the complex signal patterns resulting in great efforts to explore such effects systematically may be one reason for that.

Profound analytical studies on the variation of the original GC–MS protocol [3,4,5] mostly focused on the optimization of sample extraction [6,7,16,17], derivatization yield [18,19], and data normalization using pool samples or model experiments [7,20]. Such optimization usually aims at the maximum achievable response as optimum, based on the accurate assumption that recovery cannot exceed 100% and that the optimal protocol is the one producing the highest response. However, the response may interact not only with the probed experimental conditions but also with the composition of the sample itself, which in analytical studies is usually not modified, and sample aliquots are used instead [14].

Often, molecules of the same class occur in biological samples at concentrations differing by several orders of magnitude [21,22,23]. Thus, in plant extracts, the content of the amino acids alanine (ala), serine (ser), and glutamic acid (glu) is typically much higher than that of histidine (his) and methionine (met). The distribution of sugars is also disproportional, where glucose is much more abundant than mannose and galactose, or likewise when comparing sucrose with maltose and lactose. Moreover, if cells accumulate certain metabolites, such as osmolytes or water-soluble storage compounds such as mannitol and floridoside in many representatives of brown and red algae, respectively [23,24], these metabolites will highly dominate the metabolite profile. The same problem arises with the analysis of biological model mixtures containing certain additives in high concentrations. Other compounds may have just a very different abundance between different biological samples [16,25,26,27]. Not taking matrix effects into account during quantification in complex samples may result in serious overestimation of the metabolite content in the case of signal enhancement or in underestimation or even no detection of the analyte in the case of signal suppression.

It is textbook knowledge that the presence of matrix effects can be explored using standard addition in comparison to external calibration or by comparison of response factors to internal standards. The latter involves the addition of compounds as internal standards that are not present in the samples and co-behave with the target compounds with respect to the analytical protocol. A bias uniformly affecting the whole sample can be easily corrected by internal standards (or other simple normalization techniques such as maximum or median normalization). If the bias, however, affects individual sample composites differently, i.e., if not all target compounds in a biological extract correlate with a selected internal standard, a matrix effect of those cannot be reasonably explored, and the experiment might result in systematic errors [7,14,16,28]. In profiling analyses, many compounds, including unknowns, are typically quantified at once, and finding an appropriate number of suitable internal standards for the different compounds is a very ambitious (and possibly expensive) task [16], and specific strategies need to be developed.

Standard addition in biological extracts, on the other hand, is a very tedious method. First, the approximate concentration of the target compound in the corresponding sample must be known in order to determine the required amount of the substance to be spiked into the sample. Second, standard addition results in a large number of analyses for each sample when performed for each target compound separately. One option to make the corresponding calibration experiment more efficient would be combining standards of all target compounds in a master mix and spike the matrix with different amounts of this mix (with the precondition of paying attention to the corresponding intrinsic amount of *each* target compound). However, again, if the compounds interact with each other in different, specific ways and dependent on compound concentration, the results would finally hardly compare to the situation in the original sample, as the total molarity of the original sample would be very different to the highest concentration level (which might also apply when spiking many internal standards). In fact, even semi-quantitative comparisons may lead to erroneous conclusions in this case [16].

To determine possible solutions to avoid the occurrence of a bias or to account for its impact, one needs to understand how the particular bias disturbs accurate quantification [9]. Therefore, information on differential behavior between common target compounds for metabolite profiling is very useful for the assessment of the required robustness of the corresponding method. In this report, we compiled results from many experiments using authentic standards of native metabolites commonly detected in GC–MS profiling of biological samples to explore their matrix effects in a less complex, standard addition-type manner to identify the interaction of these compounds with each other at different concentration, within the dynamic range and saturation, during a typical experimental setup for GC–MS profiling.

## 2. Results

### 2.1. Introducing the Problem: Matrix Effects by Phosphate Observed in Glucose Analysis

**Figure 1:**
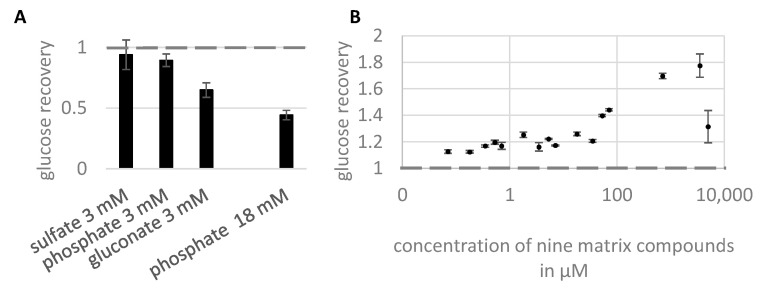
Response behavior of glucose. (**A**) Signal of 20 µM glucose in the presence of sulfate, phosphate, and gluconate at the given concentration (suppression effect); (**B**) Signal of 7 µM glucose in a mixture of model compounds (succinate, 2-oxoglutarate, phosphate, erythritol, ribose, fructose, inositol, mannitol, and sinapinic acid) in ascending concentrations (enhancement effect). Note that already for a glucose concentration of 10 µM, the enhancement effect decreased to a ratio of 1.0–1.4 in the same experiment (not shown). All experiments were conducted using a Trace Focus GC for compound separation.

In an earlier study on the quantification of carbohydrate intermediates in glycation systems [27], we noticed a critical influence of high phosphate concentration on the analysis of carbohydrates, in particular in combination with cations added to the solution, such as iron and calcium, all additives frequently applied in bioanalytical studies. The observed suppression effects prompted us to remove phosphate by solid phase extraction prior to GC–MS analysis of the carbohydrates to obtain accurate results since different phosphate concentrations were employed during this experiment. However, the same GC–MS protocol is frequently applied in our metabolite profiling work as similar protocols are applied by many colleagues in the research field, and we were interested in investigating this phenomenon in more detail. Signal decrease by cations present in high amounts was already observed and investigated earlier [26], and the presence of inorganic acid residue ions such as phosphate or sulfate was also shown to decrease the recovery of organic acids [14,26,29]. For example, Figure 1 shows the influence of two inorganic and one organic acid residue on glucose response.

Indeed, a decreased response of glucose was observed in the presence of acid residues at concentrations above 1 mM (Figure 1A). The extent of signal decrease of the target compound depended on the additive, with the closely eluting organic compound gluconic acid being the most pronounced. In a second experiment, we wanted to explore this suppression in a mixture of compounds eluting over the whole chromatogram range as a simple model of a metabolite extract (succinate, 2-oxoglutarate, phosphate, erythritol, ribose, fructose, inositol, mannitol, and sinapinic acid) with an ascending concentration between 0.07–5000 µM (~6.4 µM—45 mM total molarity). Curiously, however, during this experiment, we observed a dynamic signal enhancement of glucose (Figure 1B) up to 3 mM, which decreased towards 5 mM of each substance in the mixture. The addition of saturated phosphate again caused signal suppression of the very same compounds, including glucose (not shown). This differential behavior prompted us to explore the observed effects in more detail.

### 2.2. Matrix Effects in the GC–MS Analysis of Mixtures Containing Compounds with Similar Chemical Structure

**Figure 2:**
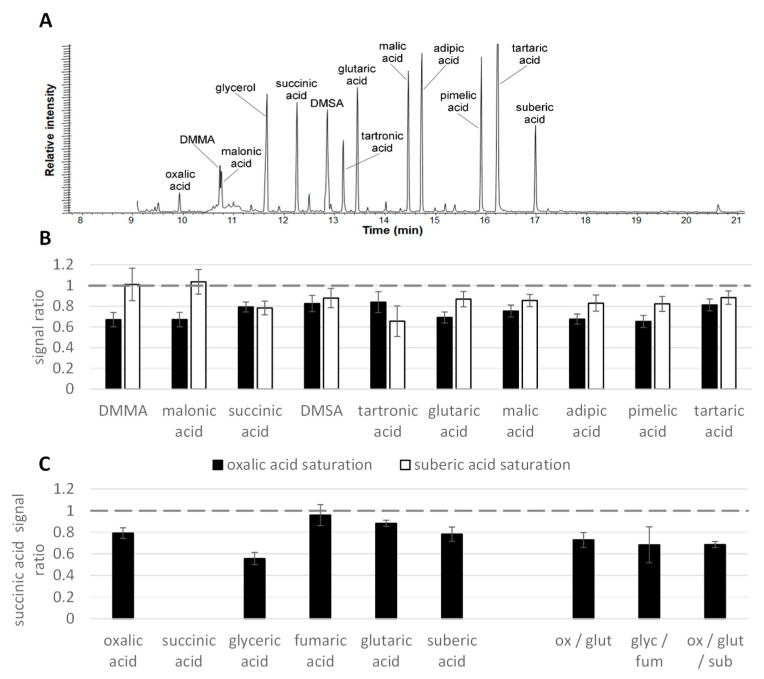
Matrix effects between organic acids. (**A**) Chromatogram illustrating the elution order of the investigated organic acids. (**B**) Recovery of ten organic acids (120 μM) in presence of oxalic or suberic acid in saturation (5 mM). DMMA—dimethylmalonic acid; DMSA—dimethylsuccinic acid. Analytes are arranged in the order of increasing retention as a proxy for decreasing volatility. Note that oxalic acid has the lowest retention, and suberic acid is the highest of the used acids. (**C**) Succinic acid (120 μM) recovery in presence of other organic acids in saturation (5 mM) as indicated on the *x*-axis. Succinic acid was included to illustrate the retention order: oxalic acid (ox) elutes ~2.4 min before succinic acid, followed by glyceric (glyc), fumaric (fum), and glutaric (glut) acids at ~0.4 min intervals and suberic acid (sub) 5 min after glutaric acid. All samples were analyzed on an Agilent GC 6890.

As a next step, we examined the response of several model compounds typical for biological samples (organic acids, sugars, and amino acids) in the presence of other substances of the same chemical class in saturation. For instance, Figure 2 shows the recovery of ten organic acids at a concentration within the dynamic range (120 μM) in the presence of 5 mM oxalic or suberic acid as the first and last eluting representatives of this compound set.

With 5 mM oxalic acid, a significant (*p* < 0.05) decrease in signal intensity was observed for all tested analytes; the level of the signal suppression varied from 16% (tartronic acid) to 35% (pimelic acid); the closely eluting malonic and dimethylmalonic acids (DMMA) were among the most suppressed analytes. Suberic acid (5 mM), though generally also tended to reduce the signal of the other organic acids, was less effective compared to more volatile oxalic acid and did not affect the signal of the most volatile tested analytes (malonic and dimethylmalonic acids).

As saturated oxalic and suberic acids demonstrated differing capacities to suppress the signals of the other organic acids, three more compounds (glutaric, glyceric, and fumaric acids) and their combinations were tested to assess a potential interaction between retention order and suppression efficiency (Figure 2C). In the example of succinic acid recovery, it was shown that the most pronounced signal suppression (by 44%) was caused by the overload of the most closely eluting compound, glyceric acid, whereas fumaric acid in the same concentration had no significant effect. Curiously, using combinations of two or three compounds (each in saturation) did not lead to a considerable increase in the suppression effect but seemed to have an intermediate effect between the single compounds in saturation (Figure 2C). In fact, both fumaric and succinic acids TMS-derivatives (C4-acids differing only in a C–C double bound) in saturation did not appear to result in any suppression of other organic acids. The derivative of glutaric acid (C5) showed only slightly stronger suppression (~12%), while oxalic (C2), glyceric (C3), and suberic acid (C8) produced suppression of >20%, indicating a differential behavior between those acids.

**Figure 3:**
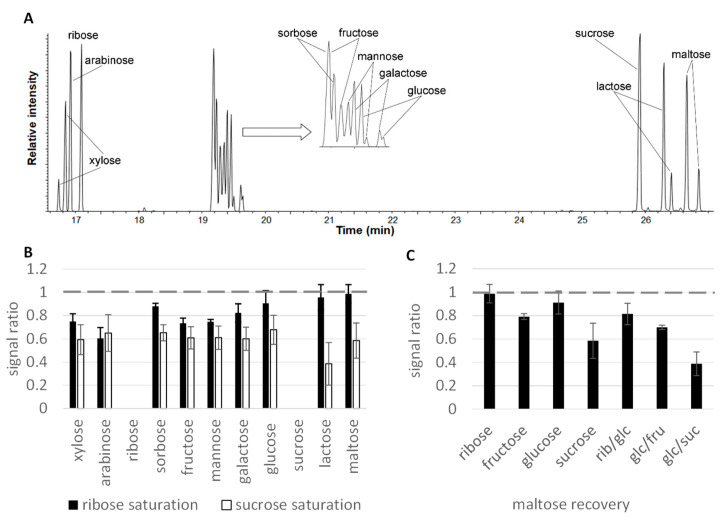
Matrix effects between sugars. (**A**) Chromatogram illustrating the elution order of the nine investigated carbohydrates. (**B**) Recovery of nine sugars (120 μM) in presence of ribose and sucrose in saturation (5 mM). Analytes are listed in order of decreasing retention, with ribose and sucrose included to illustrate the elution order. (**C**) Maltose (120 μM) recovery in presence of other sugars in saturation (5 mM). Analyzed on an Agilent GC 6890.

A second experiment was conducted on the analysis of nine mono- and disaccharides in the presence of 5 mM ribose or sucrose as early and late eluting representatives, respectively (Figure 3).

In most cases, sucrose exerted the stronger matrix effect, and the most prominent difference between ribose and sucrose effects related to the recovery of lactose and maltose, the analytes with the lowest volatility eluting just after sucrose (Figure 3B). Ribose saturation did not affect the recovery of lactose and maltose, whereas sucrose caused signal suppression by 61 and 41%, respectively. However, the closely eluting pentoses xylose and arabinose were again among the most suppressed analytes at ribose saturation. Further comparison of the impact of different sugars and their combinations (each sugar at 5 mM) on maltose recovery as the last eluting compound showed that the ability to reduce the analyte signal intensity varied considerably, and again sucrose as closely eluting peak suppressed the most (Figure 3C). Unlike a similar situation with organic acids, combinations of two sugars were generally more effective than individual compounds: the strongest suppression (by 61%) was caused by the mixture of glucose and sucrose, compounds that eluted the closest to maltose.

**Figure 4:**
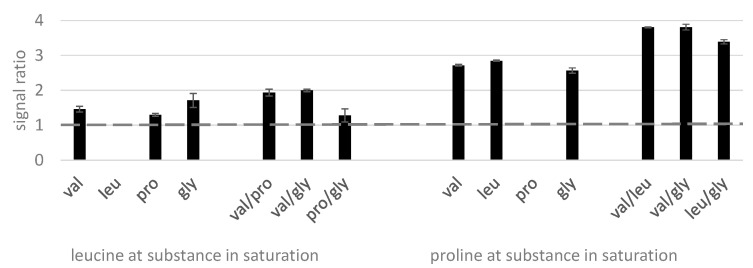
Relative recovery of the amino acids leucine and proline (350 µM) in presence of saturated valine, leucine, proline, and glycine (10 mM) and their different pairings presented in order of their retention time with leucine and proline included to illustrate the observed elution order. Analyzed on an Agilent GC 6890.

Finally, we assessed the behavior of amino acids with the model compounds valine, leucine, proline, and glycine. The obtained results are illustrated in Figure 4.

The amino acids clearly enhanced each other’s signals (Figure 4), with proline being particularly susceptible to this signal enhancement (up to 380% recovery). Here, the relative volatility of the derivative seemed less important since similar enhancement occurred with matrix compounds eluting before or after the target analyte. However, the combination of two amino acids seemed to increase signal enhancement here, with the more volatile ones being more effective. Finally, in agreement with the signal suppression behavior of the organic acids and the sugars, the amino acids eluting shortly after the overloaded substance exhibited a slightly smaller enhancement than expected, i.e., leucine at proline saturation as well as proline at glycine saturation.

### 2.3. Matrix Effects in GC–MS Analysis of Mixtures Containing Compounds of Different Chemical Natures

**Figure 5:**
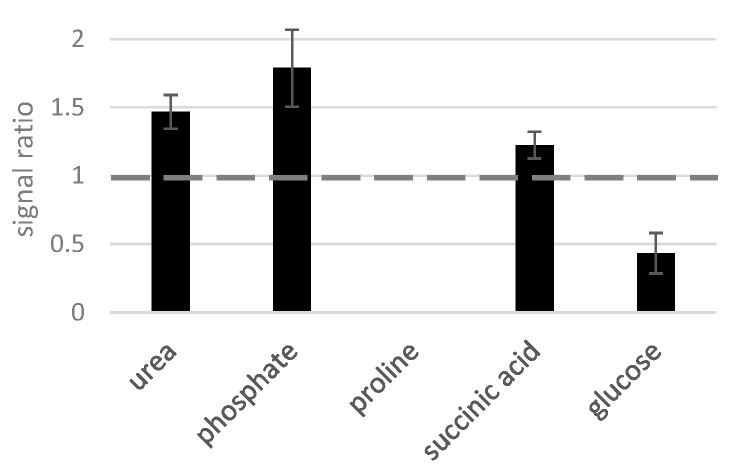
Proline (350 μM) recovery in presence of several compounds of different chemical natures in saturation (5 mM). Analyzed on an Agilent GC 6890.

As the amino acid proline demonstrated an extreme susceptibility to matrix effects (Figure 4), we chose this compound as a starting point to test mixtures of different types of compounds. In Figure 5, we present its recovery in the presence of saturated urea, phosphoric and succinic acids, and glucose.

**Figure 6:**
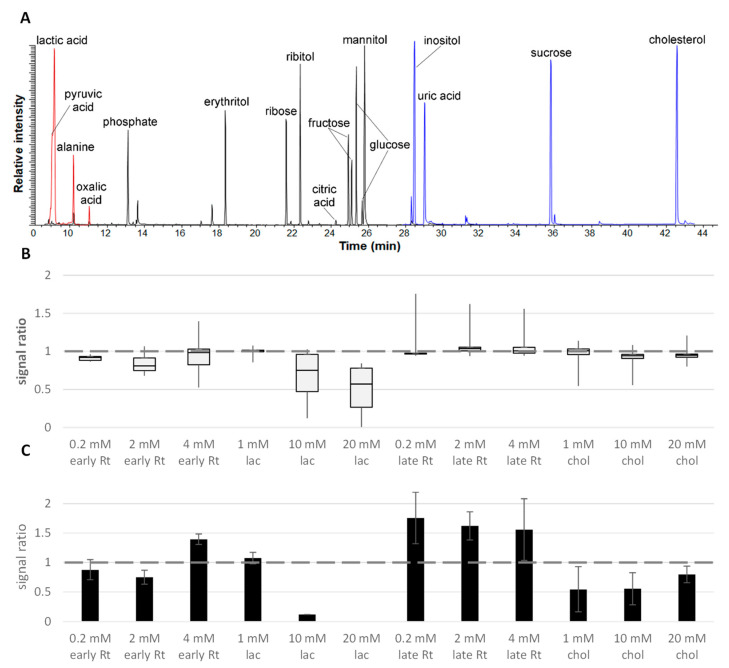
Signal behavior in presence of early and late eluting compounds in saturation. (**A**) Elution profile of the compounds used in order of retention: the early eluting derivatives of pyruvic, lactic, alanine, and oxalic acids in red (glyoxylic acid elutes within the solvent delay); phosphate, erythritol, ribose, ribitol, citric acid, fructose, glucose, and mannitol in the intermediate range (black); and four semi-volatile derivatives of inositol, uric acid, sucrose, and cholesterol (blue; a derivative of glycerophosphoglycerol was not detected; instead, degradation products such as glycerol phosphate were identified). (**B**) Boxplots illustrating signal ratio distribution for derivatives of intermediate retention (phosphate, erythritol, ribose, ribitol, citric acid, fructose, glucose, and mannitol, each 50 µM) challenged by addition of early and late eluting compounds at the stated concentration. Early eluting compounds produced a stronger suppression. (**C**) Profile of citric acid 4 TMS signal interaction with early and late eluting compounds. Lactic acid and cholesterol were suppressed, and the late eluting compound mixture enhanced the signal. Analyzed on a Trace Focus GC. Rt—retention time; lac—lactate; chol—cholesterol.

All tested substances, except for glucose, enhanced the proline’s response (Figure 5), though not to the same extent as the other amino acids (Figure 4). Curiously, the maximal proline signal (×1.8) was obtained here in the presence of saturated phosphoric acid eluting close to it; the less volatile compound succinate barely affected the proline’s response. Interestingly, although glucose elutes more than 10 min after proline, it still exhibited the strongest suppression on the amino acid. (Note that at least a similar pattern was observed for the amino compound urea as well: the signal enhancement with phosphate and/or proline was two and a half times higher than the one with glucose.) Consequently, our results imply that the volatility of the analytes derived from their elution order influence their susceptibility to matrix effects when low concentrated, and their ability to influence other analyte’s recovery when highly concentrated. To study this phenomenon in more detail, we analyzed the recovery of several compounds of medium volatility (phosphate, erythritol, ribose, ribitol, citric acid, fructose, glucose, and mannitol) in the presence of mixtures of five volatile (glyoxylic acid, pyruvic acid, lactic acid, alanine, and oxalic acid) or low-volatile substances (inositol, uric acid, sucrose, glycerophosphoglycerol, and cholesterol) in saturation. Figure 6A shows the elution profile of these substances illustrating the retention time ranges.

Considering that we previously observed the strongest matrix effect with closely eluting substances, we compared this with an experiment, where the compounds with intermediate retention times were analyzed in the presence of saturated early eluting lactate and late eluting cholesterol derivatives at the same total molarity as obtained when adding a mix of early or late eluting compounds. The results from these experiments are illustrated in Figure 6B. The recovery of most analytes decreased in the presence of early eluting compounds, whereas late eluters had only little effect. Extent and reproducibility of signal suppression depended on the concentration of the abundant compounds: while in the presence of 0.2 mM of the compound mixes (1 mM total molarity) or 1 mM single compounds, no relevant signal change was observed for all compounds, responses exhibited both a broader distribution (a more selective response change) and replicate variance at higher concentrations of matrix compounds. Moreover, we confirmed the effect of the glucose derivative, which we presented in the first section of this result part: with lactic acid in saturation, the signal of glucose reduced to 50% and 30% for 10 and 20 mM lactate, respectively, while both compound mixes did not interfere with the signal obtained at 50 µM glucose. (Note that the matrix enhancement effect shown in Figure 1 was observed to decrease for a glucose concentration above 7 µM and is no longer present at >20 µM.)

Interestingly, the single compounds, in particular lactate, produced stronger effects than the mixture of early eluting compounds (0.2 mM of 5 compounds with 1 mM total molarity vs. 1 mM single compound, 2 mM vs. 10 mM and 4 mM vs. 20 mM). Lactate considerably reduced the signal of citric acid, fructose, and glucose, whereas cholesterol just slightly (~10%) affected only citrate recovery. Indeed, fourfold substituted citric acid was the most susceptible to matrix effects (Figure 6C). Thus, citrate 4 TMS showed a large variance, especially with the addition of the late-eluting compounds (here, it was the only compound clearly above 100% recovery), but was not detected at all in the presence of 20 mM lactic acid.

Finally, in this experiment, we again observed that the signal response is affected the most for analytes in close proximity (similar retention time) to saturated compounds; for example, the erythritol signal was suppressed more by early eluting abundant compounds (down to 77%) than late eluting ones (to 92%, not shown).

### 2.4. Matrix Effects and Compound Evaporation

**Figure 7:**
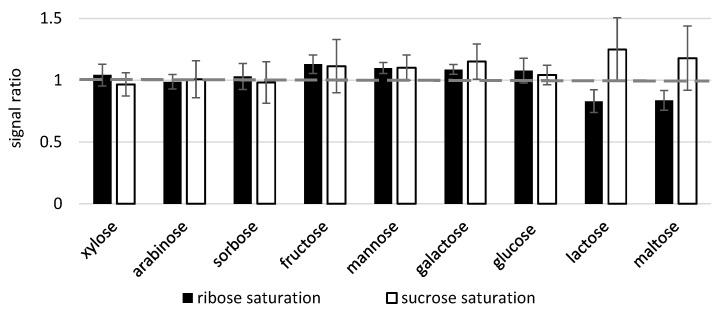
Matrix effects between sugars at a larger injection-liner volume. Recovery of nine sugars (120 μM) in presence of ribose and sucrose in saturation (5 mM). Analyzed on the Trace Focus GC.

The fact that compound volatility appears to be one of the critical characteristics for the occurrence and extent of matrix effects with our protocol suggests that processes in the injector, during evaporation and transfer of the sample to the column, maybe the main reason for such observations. Therefore, we repeated our experiment with the mono- and disaccharides described earlier (Figure 2) using a gas chromatograph with a liner of a larger volume (Figure 7).

Indeed, suppression effects observed with this repeated analysis were much less pronounced, and we considered that the larger liner volume might allow for better evaporation and transfer of the compounds. A similar effect was observed by Koek et al. [13] using a more inert, thicker film second dimension column in two-dimensional GC. Similar findings were also reported using higher oven/injection temperatures [13]. Thus, increased injector temperatures of 320 °C on the same instrument used for the experiment shown in Figure 2 also resulted in no signal suppression (data not shown), again indicating that incomplete evaporation upon addition of a compound in saturation may be responsible for the signal decrease of carbohydrates.

**Figure 8:**
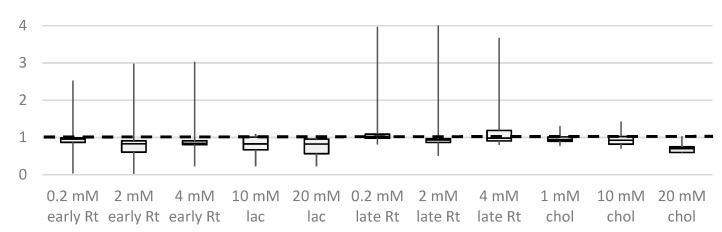
Boxplots illustrating the signal ratio distribution for eight derivatives of intermediate retention (phosphate, erythritol, ribose, ribitol, citric acid, fructose, glucose, and mannitol, each 50 µM) challenged by addition of early and late eluting compounds at the given concentration. Analyzed on a Trace Focus GC using a laminar cup splitter liner.

Incomplete evaporation may arise when the Leidenfrost phenomenon occurs in the liner region, where vapor of low boiling substances prevents the contact of the remaining liquid sample with hot surfaces. As a result, the remaining liquid sample is not transferred to the GC column but is finally removed through the split valve of the injector after the splitless time. To achieve better vaporization, we can slow the sample liquid with obstacles of a low thermal mass, such as baffles, to proceed in their path only when vaporized [30,31]. Therefore, we repeated our experiment with the different volatility groups using a laminar cup inlet liner (Figure 8). In this liner, the gaseous sample needs to pass a spiral-like, narrow channel before finally entering the column.

Compared to Figure 6B (single-tapered liner), the use of this liner clearly improved the signal behavior in the presence of abundant early-eluting compounds, including lactate. However, the trade-off was that this also seemed to cause the late eluting compound cholesterol to produce an overall signal reduction of 30% on average. Again, citric acid appeared to have a rather peculiar behavior that actually produced the large “whisker” ranges: with the laminar cup splitter, two citrate 3 TMS derivatives (which were below 5% for the single-tapered liner) were much more abundant and even increased up to 100% of the 4 TMS derivative in the reference samples (without saturated compounds) and those samples added with the late eluting compounds. In the samples added with early eluting compounds, the 4 TMS derivative was not even detected at all, and a third 3 TMS derivative was found to increase; in the samples added with 20 mM lactate, again, no derivative of citric acid was found (see Figure S1 in the Supplementary Materials). For Figure 8, the sum of the TIC area of all detected citric acid derivatives was used.

**Figure 9:**
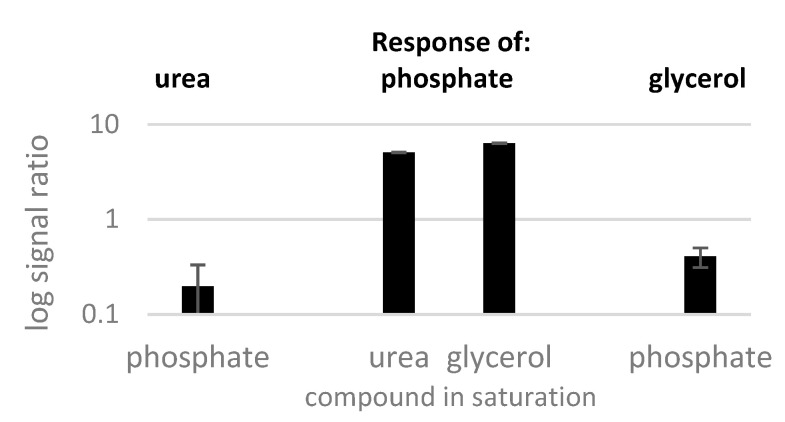
Response of 500 µM of urea, 100 µM phosphate, and 200 µM glycerol as indicated above the bars in presence of 5 mM of the saturated compound indicated on the *x*-axis, analyzed on an Agilent GC 6890.

It was reported earlier in multi-selective profiling that peaks with high intensity disturb the measurements of nearby low-concentration metabolites [12,14]. Therefore, to remove the influence of volatility (respectively elution order) and to learn if signal suppression would then be similar with different compounds, we investigated the behavior of three co-eluting derivatives of urea, phosphate, and glycerol in a second approach (Figure 9).

The co-eluting TMS derivatives of urea, phosphate, and glycerol confirmed suppression by phosphate, which in turn was enhanced by urea and glycerol. This observation finally suggests the simultaneous occurrence of specific signal suppression effects on metabolite response, here of phosphate, which might possibly relate to interference with successful derivatization [26], as also discussed in the following.

## 3. Discussion

For a joint discussion of the complex effects observed, we would first like to summarize the general trends we found during our experiments:

1. The extent of the observed effects on signal intensity was dependent on both the corresponding interacting compounds (substance-specific) and the concentration of the target and the matrix compounds. However, in our experiments with carbohydrates and organic acids, the signal was only reduced by more than a factor of ~2 when phosphate and lactate were in saturation beyond 5 mM; up to 1 mM, less suppression was observed. In fact, we found phosphate to suppress the signal of all investigated carbohydrates and organic acids at concentrations >0.1 mM in a dynamic manner. Suppression by most of the other investigated compounds as single additives started slowly only with concentrations of >0.7 mM.
2. Volatile organic acid derivatives exerted a stronger suppression on less volatile ones than vice versa. Volatile carbohydrate derivatives exerted a weaker suppression on less volatile ones than vice versa.
3. For both compound groups, organic acids and carbohydrates, the extent of matrix effects was larger among compounds exhibiting similar volatility, respectively eluting in close proximity to the compound in saturation.
4. Single compounds in saturation (e.g., lactate and cholesterol) seemed to exert stronger suppression effects than compound mixes exhibiting the same or higher total molarity.
5. The signals from derivatives of compounds with amino groups (e.g., amino acids and urea) were enhanced by more than a factor of 2 in the presence of similar compounds in saturation.

The development of accurate GC–MS methods poses serious challenges for analytical chemists considering the large range of compound classes and the large differences in concentrations within and between biological samples [14]. Moreover, in GC–MS profiling, metabolites are subjected to derivatization and might form either one derivative, two isomeric oxime–TMS derivatives, or multiple derivatives (with different response factors, mostly amines, amides, and thiols), with kinetics depending on the original metabolite sample’s composition and both contributing to the total response of the compound [32,33].

In addition, signal intensity can be compromised by interferences at all steps of the protocol [14]. Thus, within our protocol, analyte loss may occur because of the following:

- Partial evaporation during vacuum-drying (1);
- Incomplete derivatization (2);
- Incomplete evaporation during injection (3);
- Slow, respectively incomplete transfer to the GC column (4);
- Peak broadening during separation (5);
- Degradation at any step before final analysis (6);
- Inefficient ionization and ion detection in the mass spectrometer (7).

Apart from poor compound stability and degradation, during vacuum-drying (1), recovery may be influenced by the temperature during the process, the sample pH (as protonated acids usually evaporate better than the corresponding salts), and the total molarity of the sample since the vapor pressure of a compound over a sample changes with concentration. On the other hand, protons as counter-ions of acid residues were also suggested to achieve a better derivatization (2) yield during trimethylsilylation [26,29]. This could at least partly be one reason why we observed a stronger signal decrease with saturated sugars compared to organic acids, which exhibit a much favorable pKa: saturated acids more readily deliver protons to the analyte, which in turn may compensate for signal suppression by matrix background (see below) with a better derivatization yield. In return, the high suppression observed with high phosphate concentration may, in fact, also be related to the high abundance of sodium cations decreasing the derivatization yield of analytes [26]. After all, the composition of the derivatized sample dissolved in excess of reagent is a function of derivatization time until the completion of the reaction for all components of the sample [9].

After derivatization, the sample is injected (3) into the liner of a GC system, where it is supposed to be evaporated and transferred (4) to the column. At this step, different reasons for analyte loss can be assumed. Compounds that are not volatile enough for complete transfer can be retained within the injector, either in the liner, the injector body, or within the first centimeters of the column. Over time, these contaminations result in severe problems, from the formation of catalytically active sites to adsorption effects by pyrolytic particles in the liner [34]. Active sites in the liner and guard column can cause two main types of adverse interactions, i.e., chemical reactivity and adsorption. (Note that a matrix compound may also shield such active sites resulting in a signal enhancement of the analyte then). With reversible adsorption, analytes may temporarily interact with the liner surface and then slowly load onto the column, potentially leading to peak tailing or memory peaks, respectively. Irreversible adsorption, on the other hand, results in total loss of the analyte, with the analyte “sticking” in the liner. In fact, splitless injections, which are mostly used for GC–MS metabolite profiling, have lower total inlet flows, leading to longer residence times of analytes in the liner and, therefore, more time for these adverse interactions to occur. Moreover, longer residence times also subject compounds to higher temperatures in the inlet with an enhanced risk of degradation (6).

Thus, since active sites are created by the deposition of chemicals in the liner to interact with the afterward injected sample composites subsequently, we investigated liner deposition at different compound concentrations by memory peaks. Memory peaks may occur not only after compound deposition at incomplete evaporation and delayed transport to the column but also by deposited underivatized material, which is derivatized in situ and transferred during a subsequent reagent blank injection. Indeed, we found memory peaks in such subsequent blank runs starting from 0.1 mM phosphate and 0.7–0.8 mM of the other tested metabolites as single standards or starting from 0.25 mM in compound mixtures (not shown). This is a similar threshold observed for signal suppression, indicating that incomplete evaporation respectively transfers to the column might be a critical reason for the observed signal loss. Less volatile and polar substances should be more prone to retention in the liner, thus causing both losses of these analytes remaining in the liner and the formation of active sites adsorbing other compounds. Consequently, volatile analytes may be lost by adsorption to abundant less volatile compounds: incomplete evaporation of abundant high boiling compounds may lead to small droplets on which surface vaporized molecules may enter adsorption equilibrium and attach to such aggregates moving too fast through the liner to enter the column, finally leading to loss of these molecules. This could add another reason why less volatile carbohydrate derivatives exert stronger signal suppression in comparison to the more volatile organic acid derivatives. In addition, these processes may also lead to the observed signal loss in the presence of late eluting compounds such as cholesterol.

Loss of less volatile analytes during injection was already pointed out in the results part as occurring from the Leidenfrost phenomenon where vapor of abundant low boiling substances prevents the contact of the remaining liquid sample with hot surfaces [30,31]. In agreement with this hypothesis, we observed less suppressing matrix effects when using a larger liner volume or a laminar cup splitter. However, the apparently decreased derivatization grade for citric acid between a single-tapered vs. baffled liner is an adverse effect observed in our study, as the use of the latter was just expected to result in a better compound transfer. Possibly, the degradation of unstable derivatives at higher temperatures and the longer residence time in a baffled liner may be an explanation for this [35,36]. Furthermore, although the baffled liner indeed improved the signal of less volatile analytes during saturation with volatile matrix compounds, in return, it also caused a signal decrease in the presence of less volatile matrix compounds in saturation as a consequence of this improved transfer.

Signal loss during separation (5) in the GC–MS is believed to be related to analyte loss by chromatographic and *m*/*z* peak tailing, increased noise by column bleed and ghost peaks, and detection before vacuum background [37]. Thus, the column capacity for cold trapping at the beginning of the column decreases with highly concentrated analytes, and compound deposition leads to adsorptive effects causing signal suppression and higher variance. Overloading the column can produce a phenomenon at the inlet of the column, in which the saturated compound acts as a stationary phase for a sample component eluting after the major component. Some of the compounds accumulate on the back edge of the saturated compound to produce a visible peak, but an appreciable amount spreads forward under the main component peak leading to a serious underestimation of the later target compound [38].

Moreover, if components of a sample do not elute completely from the GC column and the substance remains inside the column, it may elute during subsequent analyses, sometimes very slowly and unnoticeable as an increase in noise or faster as an unexpected or overestimated peak in the chromatogram. Low volatility analytes often co-elute with the increased column bleed at the end of the temperature gradient and typically give very broad and/or tailing GC peaks or just may go undetected as slow eluting noise. Upon repeated use of GC–MS with complex extracts, vacuum background accumulates, and the noise level increases by as much as a few orders of magnitude, i.e., the MS detectability (7) of these compounds can be >10,000-fold worse than before. A plausible explanation for this is that the saturated substance cannot be pumped out as quickly, and the subsequently introduced analyte has to deal with the remainder, creating a significantly increased baseline [37]. Both effects, the matrix compound acting as a stationary phase and an increased noise level created by highly concentrated compounds, related to our observation that the response of target compounds eluting closely after a saturated matrix compound is usually affected more profoundly; the closer compounds elute in the proximity of a saturated compound [14].

Finally, we would like to suggest the decrease of matrix effects with increasing concentration of the target compound is more a consequence of a lower share of the adverse effect on the other hand, i.e., a small carry-over result in a smaller difference in peak height when higher amounts of the compound are injected [37] than of a decreasing slope at higher concentration. This effect was particularly strong for the amino acids and was also reported for amides, thiols, and sulfonic compounds [14].

The sensitive behavior of amino acids against many parameters of the standard GC–MS protocol was reported earlier in a few detailed studies on this compound group [39]. Thus, in contrast to Koek et al. [14], Noctor et al. [40] did not find any matrix effect during trimethylsilylation of sucrose, glucose, fructose, malate, and citrate (all at 2 mM) on amino acid derivatives, but they still observed a critical instability of the latter more than 2 h after derivatization. Moreover, they noticed that Asn, Gln, Ser, Thr, and Phe showed significant differences in relative abundance between standard samples and leaf extracts and suggested complex matrix effects during the injection process to be the reason for it. Kanani et al. [9] and Quero et al. [41] also suggested that the instability of some amino acid derivatives leads to high variations with time between derivatization and GC–MS analysis. Indeed, the instability of trimethylsilylated derivatives for metabolomics has long been debated, and different recommendations were concluded [14,33,35,42]. However, in our hands with GC–MS batches finished mostly after 48 h, such instability as analysis-time dependent trends in response was not observed, and repeatability was high; instead, we rather experienced a variable response pattern with those analytes between batches. Possibly, this observation may be related to the different kinetics of chemical derivatization for this compound class [32]. In fact, Kanani et al. rejected the common perception that semi-quantitative comparisons of amino acids are still valid at times shorter than the end of the silylation and suggested matrix effects as the reason [9].

Interestingly, the strong signal enhancement observed for the amino acid derivatives seemed independent of volatility, apart only from the more volatile ones exerting a stronger enhancement as matrix compounds. Possibly, with this compound group, the interaction with active sites is stronger than with each other, so the transfer to the column is greatly improved when a saturated amino acid is present as a matrix compound and shields such active sites. Another possible reason can be a higher derivatization yield when high amounts of other acids are present (note that glucose, for instance, did not enhance proline, Figure 5). Certainly, more detailed, systematic studies on the trimethylsilylation of amino acids might be useful to understand the contrasting behavior of this compound group better and to reconcile the partially contradicting observations of analytical studies on this topic.

## 4. Materials and Methods

### 4.1. Materials and Chemicals

*N*-Methyl-*N*-trifluoro acetamide (MSTFA) was purchased from Macherey–Nagel (Düren, Germany), pyridine and methoxyamine hydrochloride (MOA) were from Fluka (Buchs, Switzerland), and methanol (LC-MS grade) and sulfuric acid from Merck (Darmstadt, Germany). All other chemicals were ordered from Sigma (Taufkirchen, Germany). A laminar cup splitter liner was purchased from Restek (Bad Homburg, Germany).

### 4.2. Preparation and Use of Neat Standard Solutions

For each compound, stock solutions were prepared at 10 mM in water. For phosphate stock solution, 150 mM Na_2_HPO_4_ and NaH_2_PO_4_ were mixed, adding the acid (NaH_2_PO_4_) to the base (Na_2_HPO_4_) to adjust pH 7.4. Appropriate aliquots to achieve the desired concentration of the analytes in the derivatized sample were evaporated to dryness using an Eppendorf Concentrator 5301 centrifugal vacuum evaporator (Eppendorf, Hamburg, Germany). Linear range of all compounds was confirmed before each experiment by dilution series comprising at least seven concentration levels. A concentration from the middle of the linear range was employed for the presented experiments, as specified in the main text.

### 4.3. Experiments on Matrix Effects Caused by a High Concentration of Another Substance

A concentration of 1–20 mM was used for the analytes at high concentration/saturation in the experiments for matrix effect assessment. High concentrations produced wide chromatographic peaks with large fronting and with saturated ion signals, as observed from distortion of the isotopic patterns of abundant ions and multiplier saturation of the highest signals (Figure S2, Supplementary Materials). Different sets of analytes were chosen during our investigations, three of which contained analytes with similar functional groups (carbohydrates, organic acids, and amino acids) and mixtures containing representatives of these groups to achieve analyte sets of tailored volatility and chemical structure. All mixes containing compounds in saturation were run in triplicates. At least one blank was run after each high-concentration sample to reduce potential memory effects. Sample sequence order effects were controlled by repeated analysis of the corresponding compounds at a concentration within the linear range, which were also used as reference with 100% recovery (n ≥ 5).

### 4.4. Derivatization Prior GC–MS Analysis

Derivatization was carried out according to Hutschenreuther et al. [7]. Briefly, vacuum-dried standard mixtures were incubated by shaking in methoxyamine hydrochloride solution (20 mg mL^−1^ in pyridine) for 1.5 h at 30 °C. After that, MSTFA was added, and the samples were incubated for 30 min at 37 °C. Then, the samples were transferred to micro-inserts of GC autosampler vials (BGB, Lörrach, Germany) and subjected to GC–MS analysis.

### 4.5. GC–MS Instrumental Analysis

Two types of instruments were used for GC–MS analysis for the experiments presented here (detailed in the figure legends and the manuscript text), an Agilent 6890 gas chromatograph coupled to an Agilent MSD 5973N quadrupole mass selective detector (Agilent Technologies, Böblingen, Germany) and a Trace GC Ultra coupled with a MAT95 XP double-focusing sector field mass spectrometer (MS) (Thermo Electron, Bremen, Germany) equipped with an A200S autosampler (CTC Analytics, Zwingen, Switzerland).

Helium 5.0 (Alphagaz, Air Liquide, Düsseldorf, Germany) was used as carrier gas at 1 mL min^−1^. One microlitre (1 µL) sample was injected in splitless mode at a temperature of 250 °C and a splitless time of 90 s. While the Agilent 6890 had a liner volume of 0.9 mL, the Thermo Trace GC had a standard liner volume of 2.0 mL. The separation of analytes was accomplished on a TR-5MS column with 30 m length, 0.25 mm id, and 0.25 µm film (Thermo Electron, Dreieich, Germany). The initial oven temperature was set to 40 °C and held for 1 min, ramped with 15 °C min^−1^ to 70 °C, held for 1 min, ramped with 6 °C min^−1^ to a maximum temperature of 330 °C and finally held for 10 min. Compounds were ionized at 230 °C (HP-MSD), respectively 240 °C (sector field MS) by electron impact ionization with 70 eV and 1 mA filament current. Analyzers operated in EI full scan mode from *m*/*z* 78 to 600 with a scan time of 0.5 s per scan.

### 4.6. Data Analysis

Data were analyzed using the Xcalibur 1.4 software from Thermo Fisher Scientific (Waltham, MA, USA) based on selective mass traces for the analytes and their retention times for the GC method. When two derivatives were formed (in case of sugars, see Figure 3A), either the clearly more abundant one (e.g., glucose) or, in case of two similarly abundant ones (e.g., xylose), the sum of both, total ion current—areas were considered for evaluation. For the carbohydrate data set, fructose and sorbose were quantified based on the second derivative as the first derivatives co-eluted strongly (Figure 3A); for this, we confirmed beforehand that the ratio of the two sugar derivatives does not change under our conditions. After automated evaluation with manual curation, area tables were exported to MS Excel (Microsoft Corp., Redmont, WA, USA) for all calculations and creation of the graphs. All data are presented normalized to the response in absence of any saturated compound, i.e., the signal response of a compound within the linear range (the target compound) in presence of another, saturated compound (the matrix compound) was normalized to the response of the corresponding target compound at the same concentration from samples containing all compounds within the linear range. All analytes presented in the graphs are arranged according to their elution order as a proxy for volatility, and 100% recovery is indicated by grey dashed lines.

## 5. Conclusions

Serious matrix effects resulting in signal changes between 10% and 400% for the same concentration of a compound compromise the accuracy of profiling analyses with both semi-quantitative comparisons of non-similar profiles and quantifications of those based on only a few internal standards or external calibrations. Matrix effects appeared to depend on the target (decreasing with concentration) and matrix compound concentration (increasing with concentration). Amino compounds seemed particularly susceptible to signal enhancement (we observed signal enhancement effect up to a factor of 4), while for all other studied compounds, mostly signal suppression up to a factor of 2 occurred in the presence of saturated matrix compounds. Thus, if signal suppression is suggestive because of abundant respectively saturated signals in the chromatogram, we suggest for semi-quantitative comparisons to consider only signal changes larger than by a factor of 2; a similar recommendation was already concluded earlier for total chromatogram intensities different by more than a factor of 2 [7].

In particular, saturation with inorganic phosphate seems to impair the recovery of sugars and organic acids. Thus, to ensure the correct quantitation of these metabolites, the analyzed samples should not contain considerable amounts of phosphate [26,27]. In general, single compounds in saturation (here, lactate and cholesterol) mostly exert stronger suppression effects than compound mixtures exhibiting the same or higher total molarity, so we recommend as a minimum precaution to always check the dynamic behavior of the intensity profile of representative samples for large changes in signal abundance approaching saturation before running an experiment. If feasible, all compounds should be injected at concentrations below saturation, better below 0.7 mM, to avoid signal suppression of organic acids and carbohydrates. Alternatively, semi-quantitative comparisons between samples with very different intensity profiles, particularly with saturated compounds, may be confirmed by injecting diluted samples.

Matrix effects can be cured by improved sample preparation; however, this is often costly, time-consuming, and laborious, and thus instrument-based alternatives are highly desirable. A pooled representative of the measured samples can be used as a quality control to correct MS responses of metabolites in individual samples, as proposed by Greef et al. [43] and Kloet et al. [44], but will only work if the matrix effects do not vary between samples, i.e., when the variation of the sample composition is limited [7,14]. Unfortunately, if isotope-labeled standards are not available, only strategies such as isotope-coded derivatization or the always advisable comparison with a reference method would be able to distinguish observed changes from matrix effects [45]. As we found matrix effects often related to inefficient evaporation in the liner and impaired transfer to the column after injection, we finally recommend preventing matrix effects using a larger liner volume and/or higher temperature during the injection.

## Data Availability

The data presented in this study are available on request from the corresponding author.

## Supplementary Materials

The following supporting information can be downloaded at: https://www.mdpi.com/article/10.3390/molecules28062653/s1; Figure S1: Citrate TMS derivatives in analysis using a single tapered and baffled liner; Figure S2: Appearance of chromatographic saturation and detector overload on the example of cholesterol.

## Sample Availability

All compounds used for this project are commercially available, as indicated in the materials and methods section.
