# Supplementary material for: Matrix Effects in GC–MS Profiling of Common Metabolites after Trimethylsilyl Derivatization
Source: Molecules. 2023 Mar 15;28(6):2653. doi: 10.3390/molecules28062653 (PMC10053008; doi:10.3390/molecules28062653)
Supplement: Supplementary file 1 [file molecules-28-02653-s001.zip › molecules-2219983-supplementary.pdf]

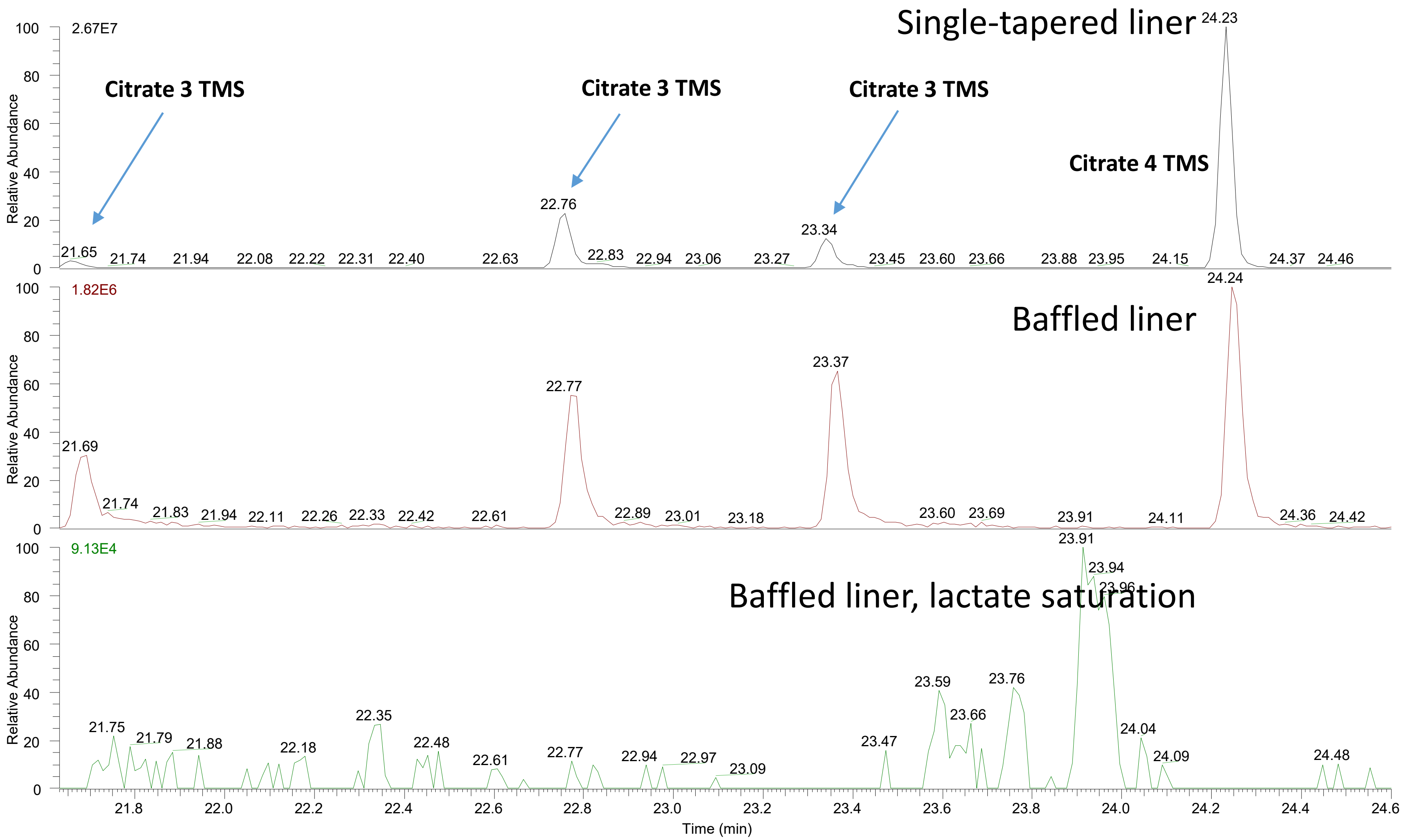

Figure S1. Selective ion chromatograms of m/z 273 specific for citrate TMS derivatives from standard mixtures analyzed with a single-tapered (top) and laminar cup splitter liner (middle and bottom). Citrate 3 TMS derivatives grew much more abundant compared to the 4 TMS derivative in the reference samples (*i.e.,* without saturated compounds) and those samples added with saturated late eluting compounds (not shown). In the samples added with 20 mM lactate, no citric acid derivative was found.

5 mM cholesterol

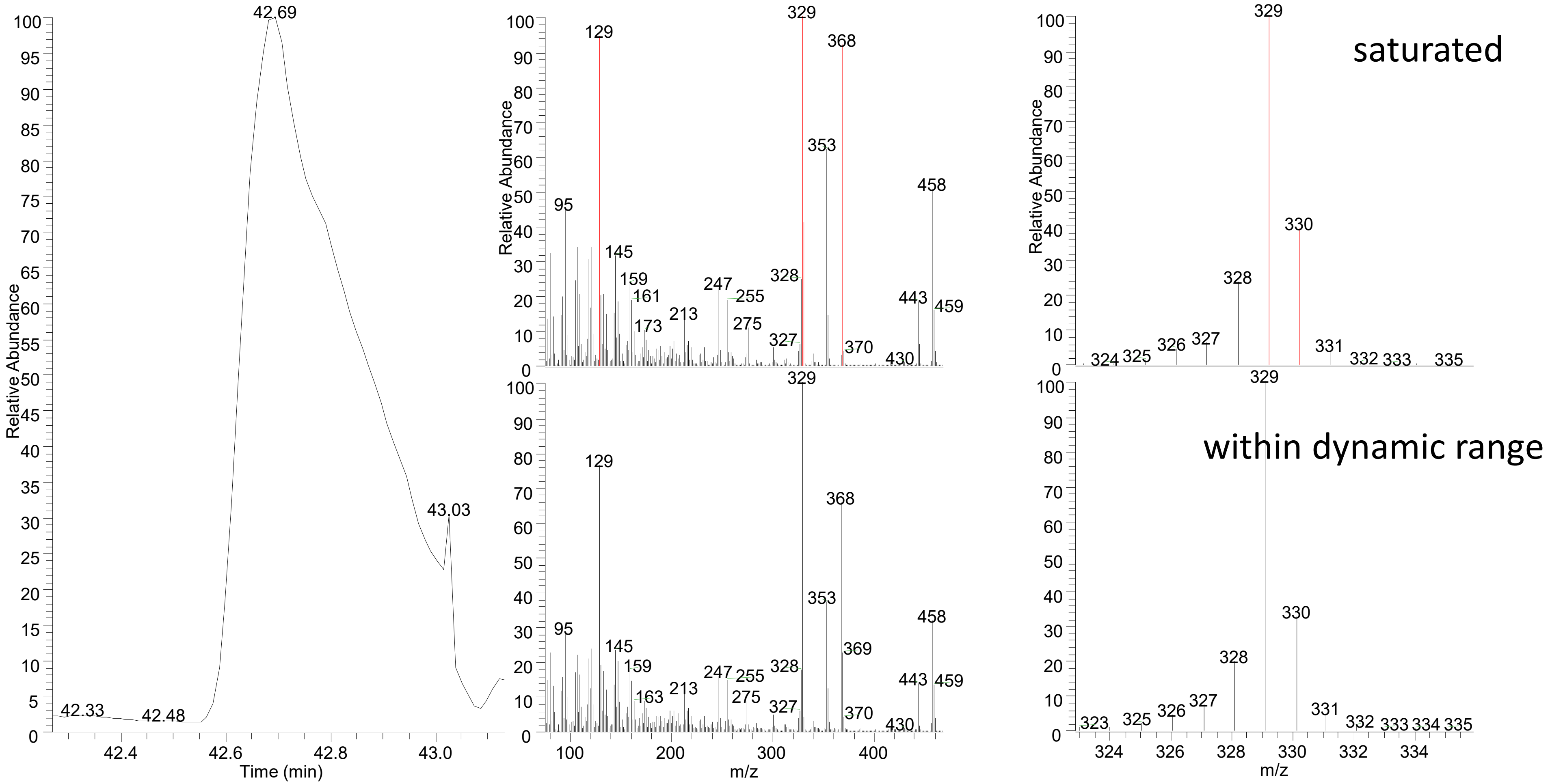

Figure S2. Appearance of compound saturation on the example of cholesterol. **Left:** chromatographic appearance was accompanied by serious peak broadening and deviation from Gaussian peak shape. **Middle:** The MS spectrum of cholesterol on the saturated apex (top spectrum, red-colored peaks are saturated) is compared with the downslope spectrum without saturated MS peaks (bottom). **Right:** A zoom into the base peak region shows the distortion of the isotopic pattern in the saturated spectrum, where peak *m/z* 330 has an intensity of ~45%, while in the downslope spectrum it is ~35%.
